# GrotUNet: a novel leaf segmentation method

**DOI:** 10.3389/fpls.2025.1378958

**Published:** 2025-07-10

**Authors:** Hongfei Deng, Bin Wen, Cheng Gu, Yingjie Fan

**Affiliations:** ^1^ Key Laboratory of Ethnic Education Informatization, Yunnan Normal University, Kunming, China; ^2^ School of Information Technology Industry, Yunnan Vocational Institute of Energy Technology, Qujing, China; ^3^ School of Information Science, Yunnan Normal University, Kunming, China

**Keywords:** instance segmentation, feature coding, jump connection, multi-scale fusion, GoogLeNet

## Abstract

In the field of biology, the current leaf segmentation method still has problems such as missed inspections and duplication in the number of large, dense, mutual obstruction and vague division tasks. The reason for the above is that image semantic extraction is not satisfactory and semantic parsing is still insufficient. To address the above problems, this paper proposes GrotUNet, a novel leaf segmentation method that can be trained end-to-end. The algorithm is reconstructed in three aspects: semantic feature coding, hopping connectivity, and multiscale upsampling fusion. The semantic coding structure consists of GRblock, WGRblock, and OTblock modules. The former two make full use of the design ideas of GoogLeNet parallel branching and Resnet residual connectivity, while the latter further mines the fine-grained semantic information distributed in the feature space on the feature map after extraction by the WGRblock module to make the feature expression richer. Unlike UNet++ dense connectivity, jump connection reconstruction only uses 1×1  convolution for feature fusion of feature maps from different network hierarchies to enrich the semantic information at each location in the space. The multi-scale upsampling fusion design mechanism incorporates higher-order feature maps into each shallow decoding sub-network, effectively mitigating the loss of semantic parsing information of feature maps. In this paper, the method is fully demonstrated on CVPPP, KOMATSUNA and MSU-PID datasets. The experimental results show that GrotUNet segmentation outperforms existential UNet, ResUNet, UNet++, Perspective + UNet and other methods. Compared with UNet++, GrotUNet improves the key evaluation metrics (SBD) by 0.57%, 0.30%, and 0.27%, respectively.

## 1 Introduction

Instance segmentation has been the most challenging task in the field of computer vision, and its techniques are widely used in the fields of intelligent driving, intelligent medical imaging, remote sensing images, and biological phenotyping. In the field of biology, the extraction and analysis of plant phenotypic features is a meaningful research work for describing organisms, which is very useful for agricultural decision-making and plant breeding industry, and helps to improve varieties and genes for plant industry, increase yield and reduce resource consumption. Currently, most plant phenotyping work is still done under field conditions by manual marking, these methods are time consuming, tedious and error prone, exploring new methods is the best way out of the dilemma. Leaf segmentation is a powerful tool for plant phenotyping. Plant leaves are characterised by various features, mainly including leaf area, leaf shape, leaf number, leaf texture, petiole and so on. Plant modal leaves have dense, mutually occluding and overlapping, large number and complex petiole joints, which makes it difficult for traditional segmentation methods to perform optimally.

The challenges of leaf segmentation and counting include two main aspects: on the one hand, challenges such as texture variations inherent to plant leaves, variations in leaf shape and size, overlap between leaves, and difficulty in distinguishing petioles. On the other hand, challenges such as variations in ambient brightness, shadows and blurring caused by wind shaking. Compared to the leaf blade, the petiole has different shapes, is very small, and exists in a very small localised area, making it difficult for some existing segmentation methods to achieve accurate segmentation. The reason is that traditional segmentation methods have insufficient extraction of fine-grained semantic features and insufficient semantic parsing reduction in the local region, which has a significant impact on segmentation performance.

In terms of feature encoding, thanks to ResNet the residual linking mechanism allows both the network layers to be deep and prevents the gradient from vanishing, evolving multiple series of architectures (He et al., 2016). Most existing segmentation architectures use it as the feature encoding extraction backbone network. GoogLeNet (Yuan et al., 2022) adopts the parallel branching idea, which allows parallel multi-branch extraction and fusion of the same input feature map, with a view to mining richer semantic information (Szegedy et al., 2015). The Outlooker neighborhood attention mechanism of the VOLO model aims at further refining the extraction of fine-grained semantics, while the Transformer can aggregate local region feature encoding to generate global contextual semantic information (Khan et al., 2022; Yuan et al., 2022). In terms of semantic parsing, UNet++ realizes the reconstruction and retention of local and global information by reconfiguring the sliding connections so that each layer carries as much local and global information as possible, and each layer is interconnected with each other, and finally shared to the last layer (Zhou et al., 2019). UNet3+ fuses the encoding module’s output feature maps with multi-scale downsampling splicing into different sub-networks of the decoding backbone (Huang et al., 2020). At the same time, the decoding high-level semantics are fused into shallow sub-networks by multi-scale up-sampling, which preserves most of the semantic information to a certain extent. Bert et al (Bert De et al., 2017). proposed discriminative loss function, which uses clustering mainly in the embedding space to recover the test instances to perform segmentation. Based on the inspirations generated by the above methods, we reconstruct the design in the feature encoding backbone network, hopping connection, and decoding backbone network, and propose a new method GrotUNet for plant leaf segmentation. The main contributions are as follows:

1. Combining GoogLeNet parallel branching with the ResNet residual join idea, the feature encoding modules GRblock and WGRblock are designed. The former is used for shallow network feature extraction and the latter for high-level feature extraction.
2. The Outlooker attention and Transformer self-attention mechanisms are introduced to further mine the fine-grained semantic information of the feature maps output from the WGRblock module, the Outlooker being used to further refine the extraction of local regions of the feature maps, while the Transformer is used to gather the attention of the nearest neighbours to generate the global contextual semantic information.
3. Reconfiguring the sliding links so that the shallow coding module outputs feature maps and the higher layer feature maps are spliced by channel after upsampling and then fused across channel features using  1×1 convolution, helping to enrich the semantic information at each location in space.
4. The decoding backbone network adopts a multi-scale up-sampling fusion design mechanism, which fuses the multi-scale up-sampling of the feature maps output from the high-level network into the shallow subnetwork to mitigate the information loss in the semantic parsing process.
5. This paper conducts comprehensive empirical studies on the CVPPP, KOMATSUNA, and MSU-PID datasets. The experimental results demonstrate that GrotUNet outperforms state-of-the-art segmentation algorithms.

The paper is organized as follows: section 2 introduces the work related to instance segmentation. Section 3 gives a detailed description of the improved GrotUNet algorithm. Section 4 provides an experimental validation of the proposed algorithm, describing the dataset, the evaluation metrics and analyzing and discussing the experimental results in detail. Section 5 gives the concluding remarks and proposes the future direction of development.

## 2 Related work

Instance segmentation is mainly categorized into candidate box extraction based and pixel classification based instance segmentation methods. He He et al (He et al., 2017). proposed the Mask R-CNN algorithm by adding a mask sub-network to the Faster R-CNN. The method connects the mask with candidate frame extraction learning and uses RoIAlign to replace RoIPooling to reduce the loss of semantic information. PANet improves the structure of the feature pyramid in the backbone network on top of the Mask R-CNN, introduces a new bottom-up pathway on the FPN, and performs aggregation between the pathways (Liu et al., 2018). DetNet introduces null convolution into the backbone framework of the network and proposes to re-train the backbone network for detection and segmentation tasks to achieve feature expressiveness and resolution (Li et al., 2018). PointRend optimizes the object edges for the up-sampling operation that get better boundary masks (Kirillov et al., 2020). RefineMask fuses more fine-grained information step-by-step in a multi-stage approach, and finally optimizes Mask R-CNN to generate rough mask edges using semantic segmentation information and edge profile information to output accurate boundary information (Zhang et al., 2021). Xue et al. (Sheng et al., 2023) improved YOLOv7 by optimizing the model structure and parameters, and then combined migration learning and optimized data enhancement methods to achieve good performance in detecting fine cigarette impurities in the stems.These methods are benchmarks for instance segmentation tasks combining target detection with target mask estimation. However, these methods become quite complex in method tuning and segmentation performance is limited when irregularly shaped targets are learned for detection. SSAP learns the pixel pair affinity pyramid. The probability of two pixels belonging to the same instance, and generates instances sequentially through cascaded graph segmentation (Gao et al., 2019). These methods generate instance masks by categorising pixels into any number of object instances in the image.

Leaf instance segmentation methods based on plant phenotypes. Romera et al. (Romera-Paredes and Torr, 2016) used LSTM network (Van Houdt et al., 2020) to train an end-to-end instance segmentation and counting network. Ren et al. (Mengye and Richard, 2017) proposed recurrent neural network combined with candidate box extraction, which showed good segmentation performance on plant leaf CVPPP dataset. Li et al. (Xingyou et al., 2024) generated pseudo defective candy images based on StyleGAN2 to enhance the negative sample data, and then background separated the color domain features of defective candies to solve the interference of the imbalance between intact and defective candy data on the detection performance. Deep Coloring simplified instance segmentation into a semantic segmentation while class labels are used for non-adjacent objects and then analyze the connected components to retrieve the instances (Kulikov et al., 2018). Victor K et al. (Kulikov and Lempitsky, 2020) proposed Harmonic algorithm which describes each object instance by using the expectation of a finite number of sinusoids and adjusts it to a specific object size and density using phase and frequency tuning. Tran et al. (Tuan et al., 2021) proposed an end-to-end reinforcement learning-based end reinforcement learning instance segmentation algorithm ColorRL. Sandesh B et al. (Bhagat et al., 2022) proposed a plant leaf segmentation algorithm Eff-UNet++. The algorithm not only adopts the lightweight Efficient-net network as the feature extraction backbone network, but also reconstructs the sliding connection part of UNet++, so that the number of parameters and the computation amount are greatly reduced. In the decoding backbone network, the high-dimensional and low-dimensional features are spliced and fused to obtain the boundary information of the object effectively. Eff-UNet++ method shows excellent performance in the dataset of plant phenotype feature segmentation.

In studies related to plant leaf segmentation, De Brabandere et al. (Bert De et al., 2017). used a discriminative loss function consisting of two parts: one part pushes the embedding means of different objects farther away from each other, and the other part pulls the embedded pixels of the same object closer to their means. The main idea is to embed the image pixels into the hidden high dimensional space, the pixels belonging to the same instance are close to each other in the space, while the pixels of different instances of the object will be embedded into different spaces, and then subsequently use clustering algorithms to generate separate instances, which is the basis of the study in this paper. In recent years, UNet architecture is widely used for segmentation tasks (Ronneberger et al., 2015). Diakogiannis et al. (Diakogiannis et al., 2020) proposed a ResUNet architecture by replacing the UNet feature extraction backbone network using a Resnet network to achieve better segmentation performance on remote sensing images. In the existing research instance segmentation end-to-end model still has more room for development. DeepLab v3+ added an effective decoder module to DeepLab v3 to recover object boundaries and achieved good performance (Chen et al., 2018). Zhou et al. (Zhou et al., 2019) proposed UNet++,interconnecting intermediate outputs between each layer with each other by means of thick connections, each module interacts with each other, and design a supervision mechanism to achieve better performance in medical image analysis. The disadvantage is that the introduction of dense connections leads to a drastic increase in the number of parameters in the model architecture, which consumes a lot of computational resources. Eff-UNet++ reduces the number of dense connections on the basis of UNet++, and fuses the high-level output feature maps into the decoding sub-networks of each layer by gradually up-sampling them, which greatly reduces the number of parameters.

Plant leaf contours, colors, and other features are very similar, coupled with the presence of occlusion and overlap between leaves, leading to tricky detection of leaf overlap and petiole regions by traditional methods. The analysis found that the network architecture of these methods causes semantic loss for feature map downsampling and upsampling. Simultaneously, there are limitations in the sensory field and insufficient ability to capture information around the spatial location of the feature map. Plant petiole features exist in a small localized area, which makes it difficult to extract such fine-grained semantic information. In order to overcome the above difficulties, this paper reconstructs the feature extraction backbone network, sliding connection, decodes the backbone network, and proposes a new plant leaf segmentation method.

## 3 Method

**Figure 1:**
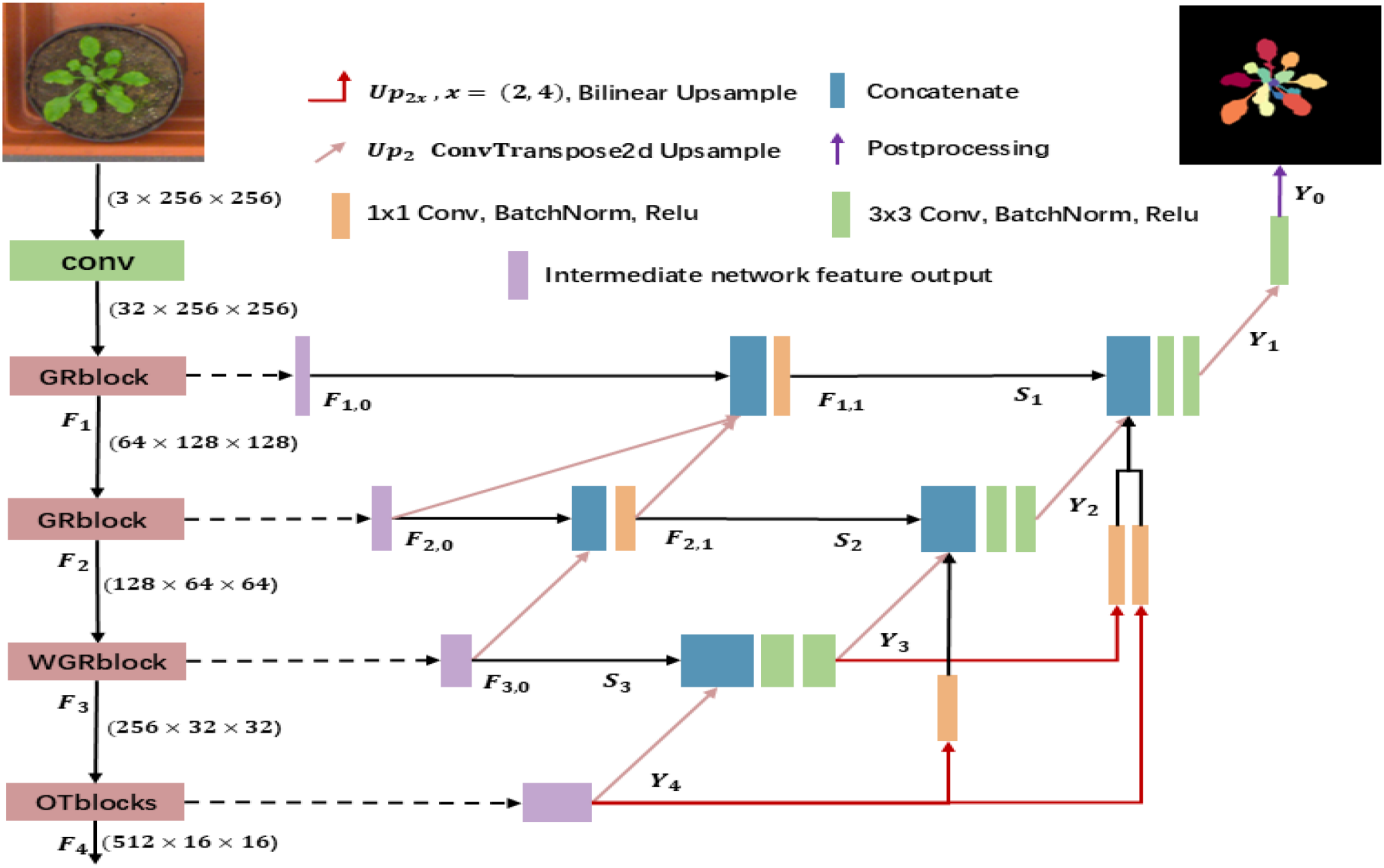
Overview of the framework.

The method proposed in this paper is shown in **Figure 1**, with the reconstructed hybrid feature coding modules GRblock, WGRblock, and OTblock on the left part, the redesigned sliding connection in the middle part, and the multiscale upsampling fusion design mechanism represented in the right part. Next, the role of each part will be elaborated in detail.

### 3.1 GROT hybrid feature encoding module

**Figure 2:**
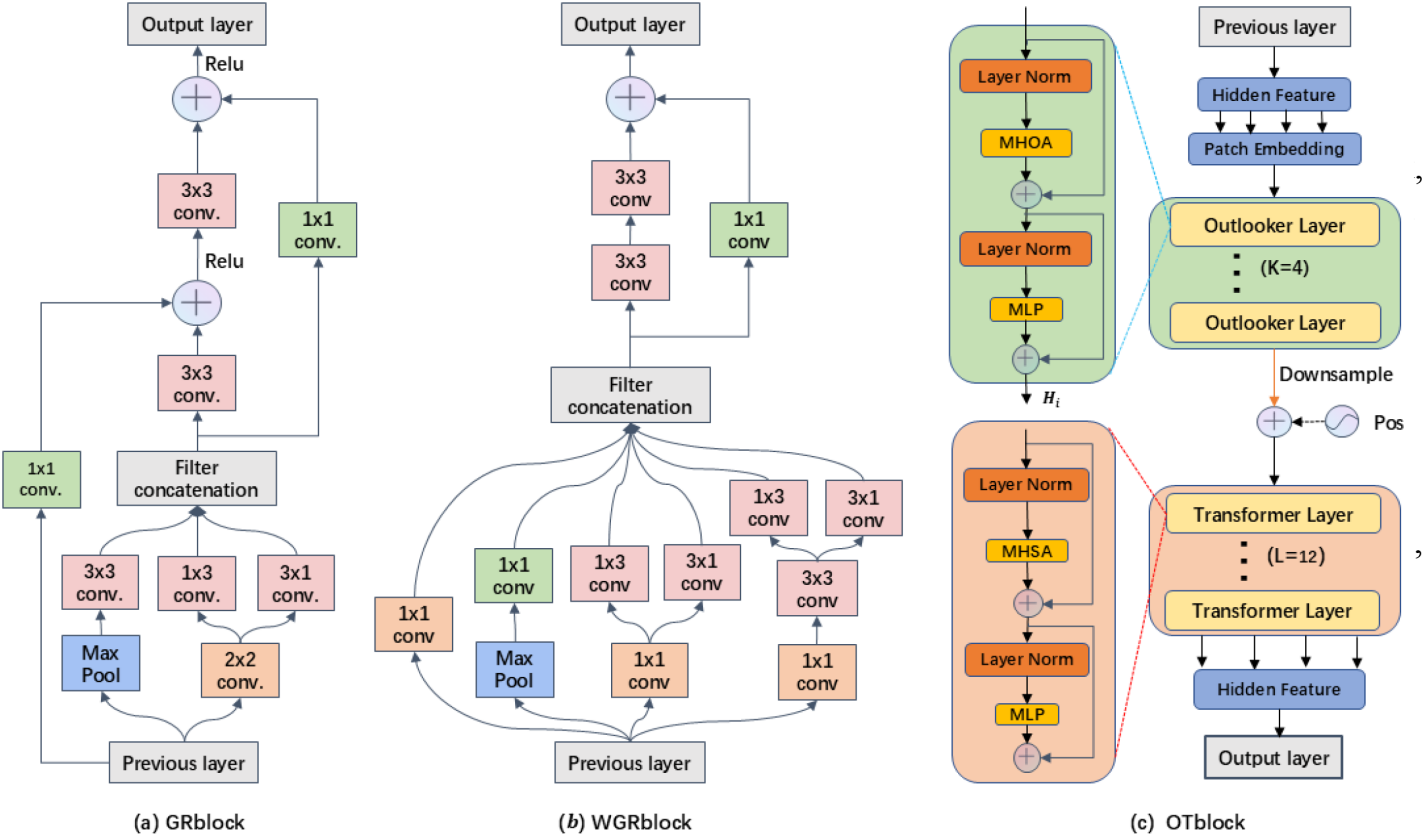
Feature extraction coding module.

The GrotUNet feature extraction backbone network consists of GRblock, WGRblock, and OTblock, as shown in **Figure 2**. Currently, the encoding backbone network of most mainstream segmentation methods is mainly based on the ResNet family of architectures. ResNet increases the depth of the network by stacking the residuals quickly, but its effective receptive field may not be as large as theoretically (He et al., 2016). Multiple Inception modules in GoogLeNet are able to capture richer feature information by applying convolution kernels of different sizes and pooling operations in parallel, but this can make the network structure relatively complex and seriously consume resources (Szegedy et al., 2015). The GRblock and WGRblock modules incorporate the parallel branching design ideas of residual block and Inception block, which can not only encode and extract different ranges of spatial feature information to enrich the feature expression, but also prevent the problem of gradient disappearance, so that the network will be more stable in the training process. The GRblock network structure is relatively simple and is mainly used to extract shallow feature maps, while the WGRblock structure is more complex and is mainly used to extract higher-order feature maps. The OTblock module is used to further extract higher-order feature maps, aiming to make fine-grained semantics further characterized. Next, the design of each coding module is described in detail.

#### 3.1.1 GRblock encoding module

GRblock is mainly used for feature extraction in low-dimensional space, and its first half uses the idea of parallel branching and the second half uses residual join, as shown in **Figure 2a**. To reduce the loss of semantic information, the module reduces the feature map size using maximum pooling and convolutional parallel two-branch downsampling with a convolutional kernel of 2. In addition, a large number of asymmetric convolutions are used in the hidden space for reducing the network computation and the number of parameters, and asymmetric convolutions are also used in the subsequent coding module. With the fast encoding and the increase of network layers, the gradient backpropagation may be decayed at each layer, which is very likely to cause the gradient vanishing problem. Residual connection is a method that can effectively solve the gradient vanishing problem, different from ResNet’s residual connection, this paper adopts the cross-residual connection to prevent the gradient vanishing problem, as shown in **Figure 2a**. Suppose the input of the module is X . The GRblock module definition **Equations 1**–**3** is shown:


(1) $$\text{ℋ}=\left[{\text{C}}_{33}\left(\text{Maxpool}\left(\text{X}\right)\right),{\text{C}}_{13}\left(C_{11}^{1}\left(\text{X}\right)\right),{\text{C}}_{31}\left({\text{C}}_{11}^{1}\left(\text{X}\right)\right)\right]$$

(2) $$\text{ℳ}=\text{Relu}\left({\text{C}}_{33}\left(\text{ℋ}\right)+{\text{C}}_{11}^{1}\left(\text{ℋ}\right)\right)$$

(3) $${\text{ℱ}}_{i}=\text{Relu}\left({\text{C}}_{33}\left(\text{ℳ}\right)+{\text{C}}_{11}\left(\text{ℋ}\right)\right)$$

Where Maxpool and $C_{11}^{1}$ represent max pooling and convolutional downsampling, respectively. $\;C_{XY}$ represents a convolution operation with a kernel of X×Y . Relu denotes the activation function.

#### 3.1.2 WGRblock encoding module

The WGRblock module structure is designed as shown in **Figure 2b**. The module uses the GoogLeNet parallel branching approach for feature extraction in different scale ranges of the input feature map, which consists of one maximum pooling branch, three stride=2 convolutional downsampling branches, and convolutional operations in tandem with each score. Maximum pooling preserves the leaf instance edge semantic information, while convolutional downsampling carries more local semantic information. Each branch, after the corresponding operation, splices and fuses the feature maps in channel direction to pass into the residual block for further feature extraction. Assuming that one of the intermediate inputs is ${ℱ}_{i}$ , The definition of WGRblock is shown as **Equations 4**–**7**:


(4) $${\text{s}}_{1}={\text{C}}_{11}^{1}\left({\text{ℱ}}_{i}\right)$$

(5) $${\text{s}}_{2}={\text{C}}_{33}\left({\text{C}}_{11}^{1}\left({\text{ℱ}}_{i}\right)\right)$$

(6) $$\text{g}=\left[{\text{C}}_{11}^{1}\left({\text{ℱ}}_{i}\right),{\text{C}}_{11}\left(\text{ℳ}\left({\text{ℱ}}_{i}\right)\right),{\text{C}}_{13}\left({\text{s}}_{1}\right),{\text{C}}_{31}\left({\text{s}}_{1}\right),{\text{C}}_{13}\left({\text{s}}_{2}\right),{\text{C}}_{31}\left({\text{s}}_{2}\right)\right]$$

(7) $${\text{ℱ}}_{\text{i}-1}=\text{Relu}\left({\text{C}}_{33}\left({\text{C}}_{33}\left(\text{g}\right)\right)+{\text{C}}_{11}\left(\text{g}\right)\right)$$

Where $C_{11}^{1}$ , ℳ represent convolution kernel 1×1 , sliding to 2 convolution and max pooling downsampling operations, respectively. $\;C_{XY}$ represents a convolution operation with a kernel of X×Y . [·] represents the splicing fusion operation by channel direction. Relu represents the activation function.

#### 3.1.3 OTblock encoding module

OTblock module consists of multiple Outlooker and Transformer attention layers, as shown in **Figure 2c**. The Outlooker neighborhood attention mechanism originates from VOLO, which was initially created to make each spatial location on the image sufficiently representative, and is designed to aggregate the attention weights of each neighboring location in the generative space, to further refine the local features. Transformer has a powerful ability to encode contextual information, and can aggregate local spatial semantic information to generate global contextual information. OTblock uses 4 Outlooker neighborhood attention layers in combination with 12 Transformer self-attention layers to further mine each location semantic information in the higher-order feature space, which is then aggregated to generate globally richer semantic information. Assume $h_{k-1}$ is the input of a layer in the middle of Outlooker, and $s_{p}^{\text{i}}$ is the intermediate data token obtained by downsampling after Outlooker extracts the neighborhood weights. The OTblock encoding definition is as shown in **Equations 8**–**12**:


(8) $${\text{h}}_{k}^{'}=\text{MHOA}\left(\text{LN}\left({\text{h}}_{\text{k}-1}\right)\right)+{\text{h}}_{\text{k}-1}$$

(9) $${\text{h}}_{k}=\text{MLP}\left(\text{LN}\left({\text{h}}_{k}^{'}\right)\right)+{\text{h}}_{k}^{'}$$

(10) $$G_{0}=\left[{\text{s}}_{p}^{1}\text{w};{\text{s}}_{p}^{2}\text{w};\ldots ;{\text{s}}_{p}^{\text{n}}\text{w};\right]+W_{\text{p}o\text{s}}$$

(11) $$G_{\text{l}}^{'}=\text{MHSA}\left(\text{LN}\left(G_{\text{l}-1}\right)\right)+G_{\text{l}-1}$$

(12) $$G_{\text{l}}=\text{MLP}\left(\text{ℒ}N\left(G_{\text{l}}^{'}\right)\right)+G_{\text{l}}^{'}$$

Where $W\in {\mathbb{R}}^{{\left(p\right)}^{2}+D}$ is the projection of the patch embedding. $W_{pos}\in {\mathbb{R}}^{N\times D}$ is the positional embedding vector, $h_{k}$ denotes the output of the k -th layer Outlooker, and $G_{l}$ denotes the output of the l -th layer Transformer. MHOA 、 MHSA 、 MLP , and LN stand for Multi-Headed Outlooker Attention, Multi-Headed Self-Attention, Multi-Layer Perceptual Machine, and Layer Normalization Operation, respectively.

### 3.2 Reconstructing Skip Connections (R-Skip)

Compared with the UNet++ sliding connection, the sliding connection reconstructed by the method in this paper reduces a large number of intermediate nodes and retains at most one node per layer, as shown in the middle part of **Figure 1**. The role of this node is mainly to aggregate the output feature maps from the current layer coding block with the feature maps output from all coding blocks and nodes of the higher layer. The purpose of using 1×1 convolution in the node is to realize cross-channel information aggregation retaining the semantic independence of each spatial location, providing rich spatial semantic features for the decoding backbone network. The definition of reconstructing the sliding connection is as shown in **Equations 13**–**15**:


(13) $$S_{3}={\text{ℱ}}_{3,0}$$

(14) $$S_{2}={\text{C}}_{11}\left(\left[{\text{ℱ}}_{2,0},U_{2}\left(S_{3}\right)\right]\right)$$

(15) $$S_{1}={\text{C}}_{11}\left(\left[{\text{ℱ}}_{1,0},U_{2}\left({\text{ℱ}}_{2,0}\right),U_{2}\left(S_{2}\right)\right]\right)$$

Where $C_{11}$ denotes the convolution kernel for 1×1 convolution operation, $U_{2}$ represents a bilinear interpolation operation with an upsampling factor of 2 . $S_{i}\;$ denotes the output of reconstructed sliding connection.

### 3.3 Multi-scale upsampling fusion decoder

In the decoding stage, the traditional method of recovering semantic information by layer-by-layer up-sampling will cause part of the semantic information to be lost, resulting in limited segmentation performance enhancement. UNet3+ designs a multi-scale up-sampling feature map fusion mechanism in the decoding backbone network, which fuses feature maps at different scales together through bilinear interpolation up-sampling splicing and aims to alleviate the loss of semantic information in the process of semantic parsing. In this paper, we reduce the number of multiscale upsampling connections on the basis of the UNet3+ decoding design, as shown in the decoding section on the right side of **Figure 1**. Assume that the output of the encoding is ${ℱ}_{4}$ , $Y_{4}={ℱ}_{4}$ . The decoding process is defined as shown in **Equations 16**–**19**:


(16) $$Y_{3}={\text{C}}_{33}\left(U_{2}\left(Y_{4}\right)\right)$$

(17) $$Y_{2}={\text{C}}_{33}\left(\left[U_{2}\left(Y_{3}\right),U_{4}\left({\text{C}}_{11}\left(Y_{4}\right)\right)\right]\right)$$

(18) $$Y_{1}={\text{C}}_{33}\left(\left[U_{2}\left(Y_{2}\right),U_{4}\left({\text{C}}_{11}\left(Y_{3}\right)\right),U_{8}\left({\text{C}}_{11}\left(Y_{4}\right)\right)\right]\right)$$

(19) $$Y_{0}={\text{C}}_{33}\left(U_{2}\left(Y_{1}\right)\right)$$

Where $C_{XY}$ denotes the operation on the corresponding convolution. $U_{d}$ represents a bilinear interpolation operation with an upsampling factor of d . [·]  stands for splicing operation by channel direction.

### 3.4 Loss functions

The discriminative loss function performs well in the field of leaf segmentation and is frequently used in many segmentation models (Bert De et al., 2017). This loss function is defined as shown in **Equations 20**–**23**:


(20) $${\text{ℒ}}_{\text{var}}=\frac{1}{C}\sum_{c=1}^{1}\frac{1}{N_{\text{c}}}\sum_{\text{i}=1}^{N_{\text{c}}}{\left[∥{\text{μ}}_{c}-{\text{x}}_{i}∥-{\text{δ}}_{v}\right]}_{+}^{2}$$

(21) $${\text{ℒ}}_{dist}=\frac{1}{C\left(C-1\right)}\sum_{c_{A}=1}^{C}\sum_{c_{\text{ℬ}}=1}^{C}{\left[2{\text{δ}}_{d}-∥\mu_{cA}-\mu_{c\text{ℬ}}∥\right]}_{+}^{2},\;\left(c_{A}\neq c_{\text{ℬ}}\right)$$

(22) $${\text{ℒ}}_{\text{reg}}=\frac{1}{C}\sum_{\text{c}=1}^{C}∥{\text{μ}}_{c}∥$$

(23) $$\text{ℒ}=\text{α}\cdot {\text{ℒ}}_{\text{var}}+\text{β}\cdot {\text{ℒ}}_{\text{dist}}+\text{γ}\cdot {\text{ℒ}}_{\text{reg}}$$

Where C denotes the number of instances of the real labeled image, $N_{c}$ denotes the number of pixels in a particular instance C , $x_{i}$ denotes the i -th pixel in the instance that generates the embedding vector, and $\mu_{c}$ is the mean vector of the real labeled instances  C , which represents the clustering center. ∥·∥ is the ${ℒ}_{1}$ or ${ℒ}_{2}$ distance, which represents the canonical term. $\delta_{v}$ and $\delta_{v}$ represent the variance and distance of the margins, respectively. The discriminative loss function aims to bring the embedding vectors of the pixels inside the same instance as close as possible to the center of the mean value of that instance in the mapping space. The mean vectors of different instances are to be as far away from that mean center as possible.

## 4 Experiments and analysis

This section will detail the experimental design and analysis. Firstly, the different characteristics of the three datasets are briefly introduced. Secondly, the evaluation metrics for instance segmentation leaf segmentation are presented. Then, some details of the algorithm training configuration are presented. Finally, the performance of the proposed method is evaluated and compared with state-of-the-art methods.

### 4.1 Dataset and evaluation metrics

#### 4.1.1 Dataset

In the process of experimental demonstration, three datasets, CVPPP, KOMATSUNA, and MSU-PID, are selected to verify the effectiveness and segmentation performance of GrotUNet. Next, the reasons and circumstances of data set selection are described in detail.

Due to the small data volume of the CVPPP A1 dataset, the blade contour is clear and the labeled file contour is delicate, which can effectively test the performance of the algorithm (Minervini et al., 2016). Therefore, it is often used as a benchmark evaluation dataset for mainstream instance segmentation methods.A1 contains a total of 161 leaf images, 128 in the training set and 33 in the test set. In order to better evaluate the performance of the algorithm, in the experiments, the training set A1 is divided into 85% for training and 15% for validation, i.e., 108 sheets are used for model training and 20 sheets are used for evaluating the qualitative test.

The KOMATSUNA leaf dataset acquisition was done at 4-hourly intervals under an ambient condition of illumination of 2400 lua, temperature of 30°C, and humidity of 30%, with a total of 900 images (Uchiyama et al., 2017). The moderate data volume of KOMATSUNA, along with the low number of leaves per image, facilitates the observation of segmentation of details such as petiole and helps in the validation of the model.The KOMATSUNA dataset is divided into 80% training dataset and 20% testing dataset, i.e., 720 images for training the model and 180 images for evaluation testing.

MSU-PID is the first multimodal plant image database, which contains two kinds of plants, Arabidopsis and Bean (Cruz et al., 2016). The Arabidopsis data contains four modalities, 2160 RGB modal images and 576 labeled images. The leaf overlap of the Arabidopsis data is blurred, which is difficult to distinguish, and it is more testing for the performance of the model. In the experiment, the Arabidopsis data was preprocessed to collect 576 source data that corresponded one-to-one with the labeled images, which were divided into 80% for the training set and 20% for the testing set, i.e., 460 for training and 116 for evaluation testing.

The method proposed in this paper uses data augmentation techniques to expand the training set prior to training, including random cropping, random up and down flipping, and random left and right flipping.

#### 4.1.2 Evaluation metrics

For the evaluation metrics to evaluate the model segmentation performance,  FBD , SBD are used. the evaluation metrics for the number of instances, DiC , |DiC| are used, and the details of each metric are introduced as follows:

Foreground Background Dice (FBD) is the foreground mask dice coefficient (Scharr et al., 2016). It mainly measures the degree of overlap between the real labeling $P^{gt}$ and the background binary segmentation mask of the algorithm’s prediction result $P^{pre}$ . It is used to evaluate the ability of the algorithm to recognize the target from the background and perform binary segmentation. FBD is defined as shown in (Diakogiannis et al., 2020):


(24) $$FBD\left(\%\right)=\frac{2\left|P^{gt}\cap^{}P^{pre}\right|}{\left|P^{gt}\right|+\left|P^{pre}\right|}$$

Symmetric Best Dice (SBD) denotes the average Dice between all the instances (Scharr et al., 2016). Each predicted label produces dice with the real label and then averages them to estimate the average instance segmentation accuracy. BD is defined as follows:


(25) $$BD\left(L^{a},\;L^{b}\right)=\frac{1}{M}\sum_{i=1}^{M}\underset{1\leq j\leq N}{\max}\frac{2\left|L_{i}^{a}\cap L_{\text{j}}^{b}\right|}{\left|L_{i}^{a}\right|+\left|L_{j}^{b}\right|}\;$$

|·| denotes the number of pixels. $L_{i}^{a}\;\left(1\leq i\leq M\right)和L_{j}^{b}\left(1\leq j\leq N\right)$ belong to the segmentation sets $L^{a},\;L^{b}$ respectively.

The SBD of the true labeled set $L^{gt}$ and the predicted labeled set $L^{pre}$ is defined as follows:


(26) $$SBD\left(L^{gt},L^{pre}\right)=\min\left\{BD\left(L^{gt},L^{pre}\right),\;BD\left(L^{pre},L^{gt}\right)\right\}$$

Difference in Count (DiC) represents a measure of the difference between the predicted number of instances and the true number of instances (Scharr et al., 2016). |DiC| is the absolute value of DiC. DiC is defined as follows:


(27) $$DiC=\#L^{pre}-\#L^{gt}\;$$

### 4.2 Experimental details

The experimental demonstration is mainly based on the deep learning framework PyTorch, and the specific environment configuration and parameter settings are shown in **Table 1**. The parameter α , β , γ  value settings in the loss function are consistent with the study of Bert et al. (Bert De et al., 2017). The optimizer selects AdamW, and the weight decay is 0.05. The initial value of the learning rate is set to 0.001, and the decay factor is 0.1. In order to verify the validity and generality of the model the proposed method in this paper, the hyperparameters are configured identically during the training of the three datasets, CVPPP, KOMATSUNA and MSU-PID. The image sizes of the model inputs are 256×256×3, 256×256×3 , and 128×128×3 , respectively.

**Table 1:** Environment configuration and parameter configuration during experiment implementation.

Experimental setting	Configurations	Parameter setting	Configurations
Operating system	Ubuntu20.04	Batch size	16
CPU	12 vCPU Intel(R) Xeon(R) Platinum 8352V CPU @ 2.10GHz	epochs	200
GPU	vGPU-32GB(32GB) * 1	Ir	1-e3
CUDA Versions	CUDA 11.3	Weight decay	5-e2
Python Edition	Python 3.8	α	1
Deep Learning framework	PyTorch	β	1
Torch versions	1.10.0	γ	0.001

### 4.3 Experimental analysis and discussion

The method in this paper evaluates the segmentation performance through two dimensions: the intuitive visual perception of the visualization effect and the instance segmentation evaluation metrics. The training and test sets were kept constant during the experimental implementation and were trained and tested independently at CVPPP, KOMATSUNA and MSU-PID using the same parameter settings.

#### 4.3.1 Comparison of state-of-the-art methods

To verify the segmentation performance of the GrotUNet model on plant leaves, this paper carries out experimental comparisons using six state-of-the-art segmentation methods, namely UNet (Ronneberger et al., 2015), ResUNet (Diakogiannis et al., 2020), UNet++ (Zhou et al., 2019), DeepLab V3 (Chen et al., 2018), DSNet (Guo et al., 2024), and Perspective + UNet (Hu et al., 2024). All methods keep the same parameter settings and loss functions during the experiment. The results of the evaluation of this paper’s methods on CVPPP, KOMATSUN and MSU-PID datasets are given in **Tables 2**, **3** and **4**, respectively. It is observed that GrotUNet achieves FgBgDice: 98.07, 97.80, 91.20; SBD: 89.50, 92.44, 85.48 for the leaf segmentation evaluation metrics on the three datasets, respectively.Meanwhile, the counting evaluation metrics on the three datasets achieves DiC: 0.05, -0.07, -0.06; |DiC|. 0.55, 0.16, 0.28. In terms of the key segmentation evaluation metrics FBD and SBD evaluation, GrotUNet performs the best on all three datasets, exhibiting strong segmentation performance.

**Table 2:** Comparison results with state-of-the-art methods on the CVPPP dataset.

Method	Flops(G)	Parms(M)	FPS	FBD(%)	SBD(%)	DiC	|DiC|
UNet (Ronneberger et al., 2015)	43.45	14.02	**177.72**	96.80	82.67	-0.1	0.8
ResUNet (Diakogiannis et al., 2020)	23.87	69.31	104.84	96.68	86.51	-0.1	0.6
UNet++ (Zhou et al., 2019)	200.96	47.20	84.75	98.36	88.93	0.05	0.55
DeepLabv3+ (Chen et al., 2018)	**7.79**	**5.86**	117.38	96.63	84.97	0.1	0.7
DSNet (Guo et al., 2024)	33.03	29.33	51.19	93.21	73.90	0.1	0.7
Perspective + UNet (Hu et al., 2024)	90.49	103.85	39.83	97.98	87.86	0.25	0.75
GrotUNet	47.99	104.45	36.82	**98.07**	**89.50**	**0.05**	**0.55**

**Table 3:** Comparison results with state-of-the-art methods on the KOMATSUNA dataset.

Method	FBD(%)	SBD(%)	DiC	|DiC|
UNet (Ronneberger et al., 2015)	96.58	83.71	0.23	0.47
ResUNet (Diakogiannis et al., 2020)	96.39	88.54	**-0.04**	0.28
UNet++ (Zhou et al., 2019)	97.90	92.14	-0.05	0.20
DeepLabv3+ (Chen et al., 2018)	96.85	86.78	-0.1	0.39
DSNet (Guo et al., 2024)	93.88	80.78	0.05	0.38
Perspective + UNet (Hu et al., 2024)	97.58	92.15	**-0.04**	**0.16**
GrotUNet	**97.80**	**92.44**	-0.07	**0.16**

**Table 4:** Comparison results with state-of-the-art methods on MSU-PID dataset.

Method	FBD(%)	SBD(%)	DiC	|DiC|
UNet (Ronneberger et al., 2015)	88.84	80.81	-0.13	0.53
ResUNet (Diakogiannis et al., 2020)	91.11	84.76	-0.12	0.35
UNet++ (Zhou et al., 2019)	91.00	85.21	-0.17	0.35
DeepLabv3+ (Chen et al., 2018)	90.23	82.38	**-0.01**	0.31
DSNet (Guo et al., 2024)	87.16	75.36	-0.29	0.58
Perspective + UNet (Hu et al., 2024)	90.83	84.67	**0.01**	**0.26**
GrotUNet	**91.20**	**85.48**	-0.06	0.28

**Figure 3:**
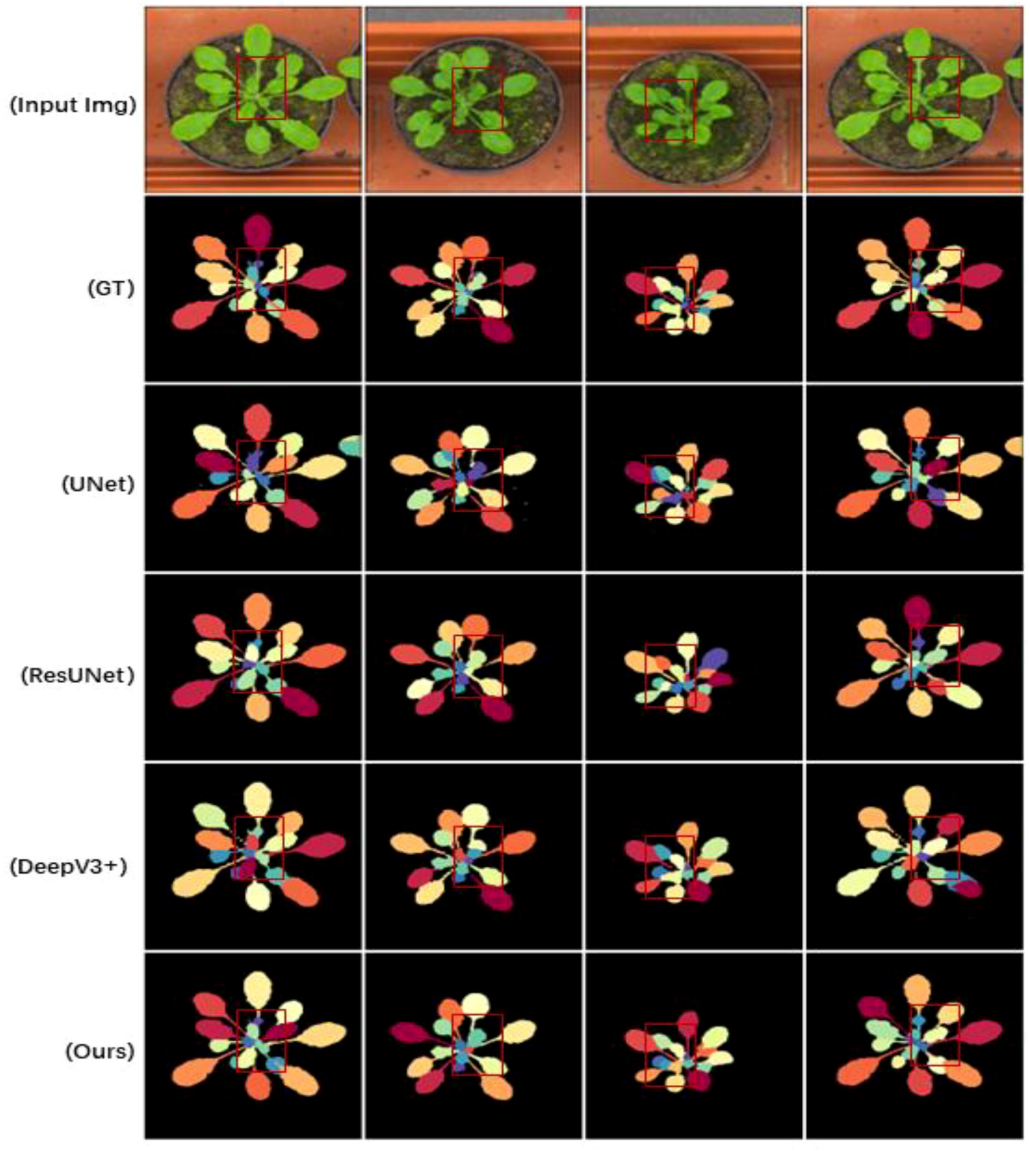
Visualizing sample results on the CVPPP dataset.

**Figure 4:**
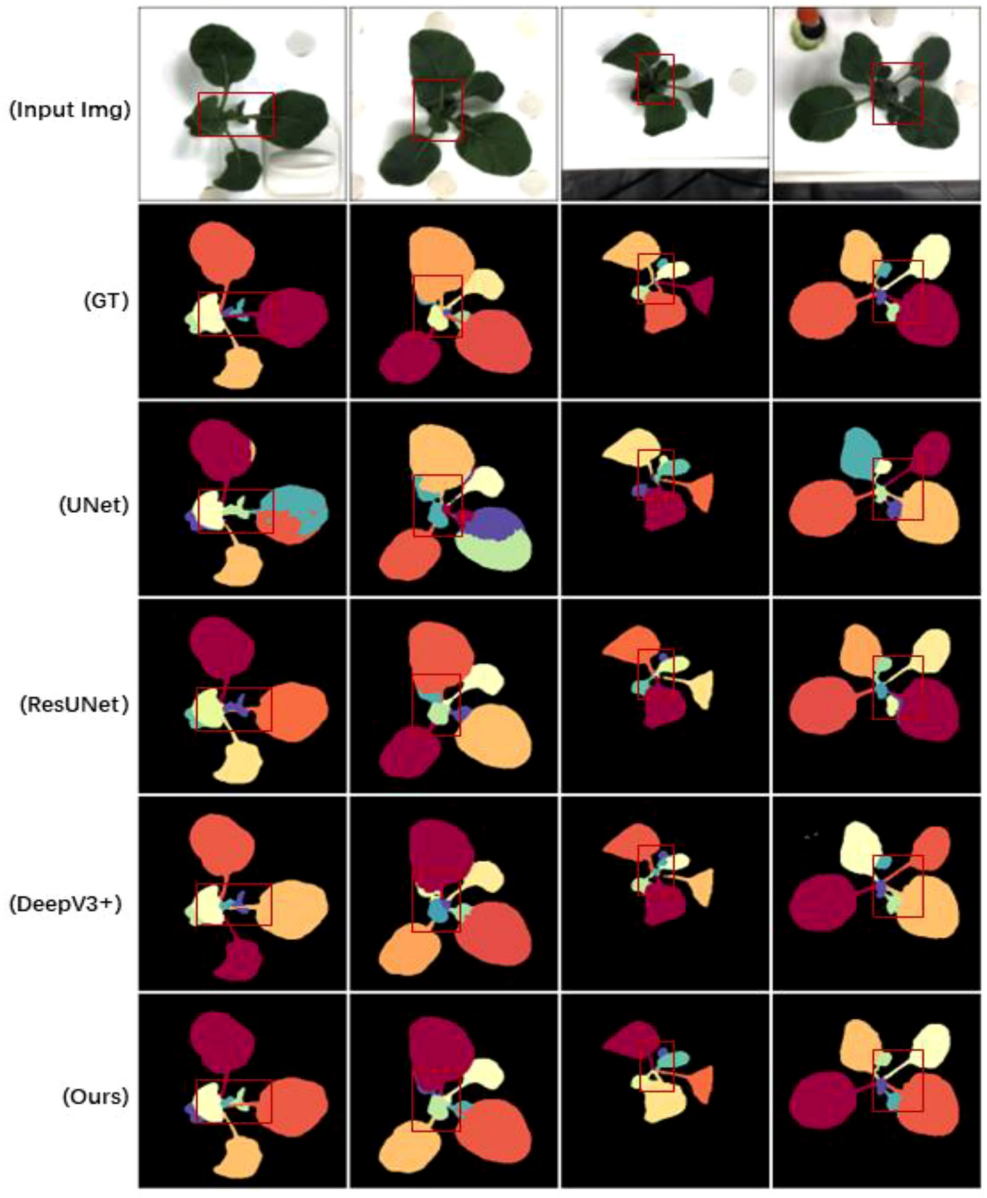
Visualizing sample results on the KOMATSUNA dataset.

**Figure 5:**
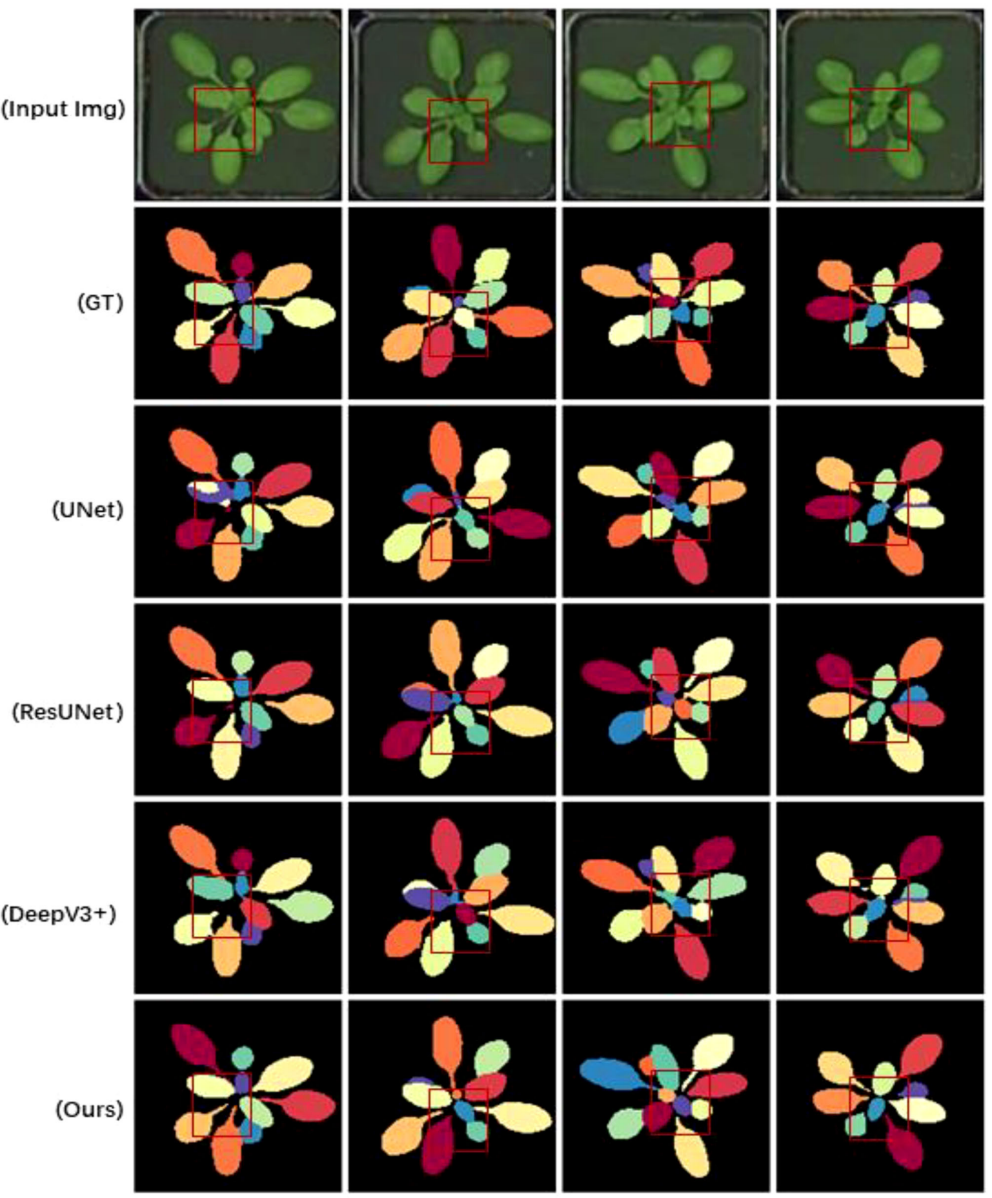
Visualizing sample results on the MSU-PID dataset.

**Table 2** demonstrates the results of Flops, Parms, and FPS comparisons, where GrotUNet has a large number of parameters and is too slow for inference, but the computational complexity is better than UNet++. **Figures 3**, **4**, and **5** show the visualized qualitative results of GrotUNet compared with several other state-of-the-art methods on CVPPP, KOMATSUN, and MSU-PID datasets, respectively. Observing the three visualizations, it is easy to find that the segmentation of petiole aggregation region by UNet, ResUNet, and DeepV3+ methods on the three plant datasets is unsatisfactory, and some petiole features are not captured. In addition, at the overlap of petiole and leaf blade, and at the boundary between petioles, the ability of GrotUNet to capture fine-grained semantics in the local area is significantly better than that of UNet, ResUNet, and DeepV3+, and the fine features such as petiole are almost completely recognized. Traditional segmentation methods in feature map scale transformation downsampling and upsampling will cause some key semantic information to be lost. Furthermore, the range of sensing field is more limited, and richer features cannot be acquired. The method proposed in this paper prevents the loss of semantic information during the flow of image semantics through the reconstruction of the feature extraction backbone network, sliding connection and multiscale up-sampling fusion mechanism in order to prevent the loss of semantic information during the flow of image semantics in the feature layer.

#### 4.3.2 Comparison with existing studies

To validate the performance of GrotUNet for further verification, this paper compares the evaluation results on CVPPP, KOMATSUNA, and MSU-PID datasets with the extant research methods. **Table 5** demonstrates the SBD, |DiC| comparison results on the CVPPP dataset. **Table 6** gives the results of comparison between KOMATSUNA and MSU-PID datasets on SBD. Comparing the 2 tables, the performance of GrotUNet leaf segmentation is better than the existing research methods. IPK performs poorly in leaf segmentation and counting due to overlapping leaf blades and crossing leaf margins (Pape and Klukas, 2015). The presence of small leaves and petioles resulted in reduced segmentation and detection ability of Nottingham and Wageningen (Yin et al., 2014a; Scharr et al., 2016). The poor segmentation ability of MSU may be due to dense leaves (Scharr et al., 2016). Deep coloring may have too many post-processing hyper-parameters, which resulted in limited segmentation ability (Kulikov et al., 2018). The method proposed in this paper combines the discriminative loss function to reconstruct the feature extraction backbone network, the sliding connection, and the decoding backbone network, and the performance in leaf segmentation and leaf number calculation reaches the advanced level.

**Table 5:** Comparison of SBD and |DiC| results between GrotUNet and state-of-the-art methods on CVPPP. Dataset.

Method	|DiC|	SBD (%)
IPK (Pape and Klukas, 2015)	2.6	74.4
Nottingham (Hu et al., 2024)	3.8	68.3
MSU (Hu et al., 2024)	2.3	66.7
Wageningen (Yin et al., 2014a)	2.2	71.1
Recurrent IS+CRF (Romera-Paredes and Torr, 2016)	1.1	66.6
E2E (Mengye and Richard, 2017)	0.8	84.9
DLoss (Bert De et al., 2017)	1.0	84.2
Deep coloring (Kulikov et al., 2018)	2.0	80.4
ColorRL (Tuan et al., 2021)	1.34	87.3
Eff-UNet++ (Bhagat et al., 2022)	1.15	85.0
GrotUNet	**0.55**	**89.5**

**Table 6:** Comparison of SBD results between GrotUNet and advanced methods on KOMATSUNA and MSU-PID Dataset.

KOMATSUNA		MSU-PID	
Method	SBD(%)	Method	SBD(%)
CVPPP-All (Ward and Moghadam, 2020)	51.34	(Yin et al., 2014a).	63.0
Ward et al (Ward et al., 2018).	62.43	(Yin et al., 2014b).	64.4
UPGen (Ward and Moghadam, 2020)	71.69	(Yin et al., 2017).	65.2
Upen-Incontext (Ward and Moghadam, 2020)	77.76	(Yin et al., 2017).	61.0
Eff-UNet++ (Bhagat et al., 2022)	83.44	Eff-UNet++ (Bhagat et al., 2022)	71.17
GrotUNet	**92.44**	GrotUNet	**85.48**

As far as the network architecture is concerned, traditional encoding-decoding architectures lose some of the semantic information in both image downsampling and upsampling. UNet++ improves segmentation performance by constructing sliding connections in the form of dense connections, which, however, imposes a large amount of computation. Compared with UNet++, GrotUNet’s sliding connection design drastically reduces the number of nodes, computational effort, and number of parameters, and retains sufficient semantic information. The multi-scale upsampling fusion design mechanism fuses the higher-order feature maps into the lower-order sub-networks while using 1×1 convolution for feature aggregation. This not only balances the excessive number of parameters well, but also mitigates the semantic loss in the decoding process.

**Figure 6:**
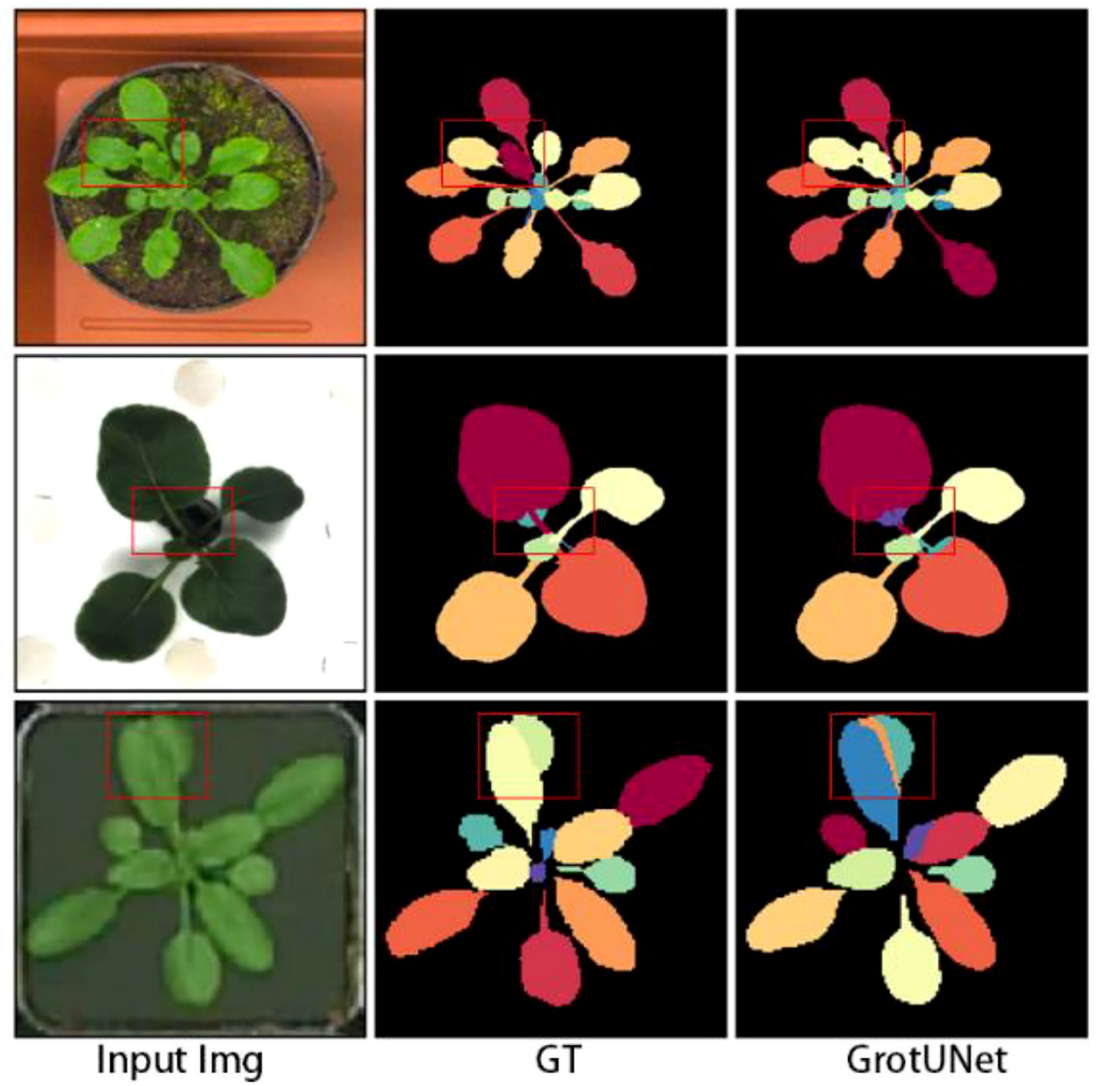
Failed cases of GrotUNet segmentation on CVPPP, KOMATSUNA and MSU-PID datasets.

Some cases of segmentation failure of GrotUNet on CVPPP, KOMATSUNA and MSU-PID datasets are shown in **Figure 6**. It is observed that GrotUNet is unable to accurately detect the number of plant leaves, leaf edge contour, petiole region, etc., and is not sensitive enough to the local area features, which leads to some incorrect segmentation. Meanwhile, this also restricts the further improvement of the model performance, and further research will be done subsequently.

### 4.4 Ablation study

The existing backbone network for feature extraction in segmentation methods mainly uses the ResNet family of architectures, but in the experiments, it is found that these mainstream architectures perform poorly for detail semantic extraction such as petiole, which restricts the performance of the model. Analyzing the reasons, it may be found that the ResNet architecture itself is deficient in the presence of insufficient feature extraction and serious loss of detail semantics when the feature map scale is reduced. Based on this, this paper redesigns the hybrid feature extraction backbone network, and the ablation study is mainly carried out on this basis. The ablation study is carried out on CVPPP, KOMATSUNA and MSU-PID datasets, and the experimental ablation study mainly verifies the performance of R-Skip, Muti-UP, and OTblock modular design on the key evaluation index SBD, so as to verify whether each module contributes to the segmentation performance.

**Figure 7:**
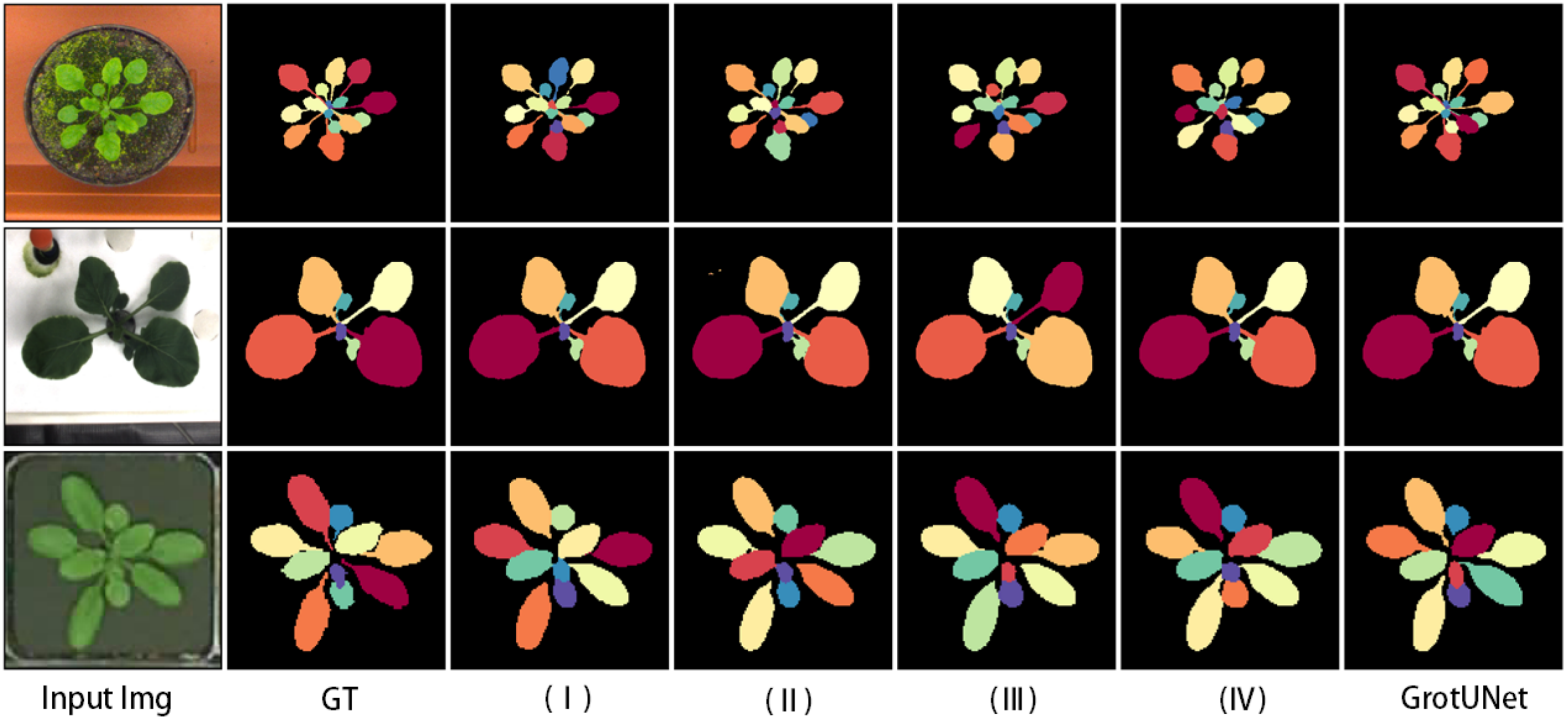
Ablation study visualization results on CVPPP, KOMATSUNA and MSU-PID datasets.

**Table 7** and **Figure 7** give the results and visualizations evaluated on the CVPPP, KOMATSUNA and MSU-PID datasets. Observation of the graphs reveals that Configuration II, which uses the same sliding method and decoding structure as UNet and does not employ the R-Skip and Muti-UP modules, performs poorly in segmenting the petiole detail region. Configurations I, III, and IV have different leaf segmentation performances on the CVPPP, KOMATSUNA, and MSU-PID datasets, and the segmentation effect is unsatisfactory in detail regions such as petiole. However, when R-Skip, Muti-UP, and Otblock modules are all applied, the best leaf segmentation performance is realized and SBD is improved significantly. In addition, **Table 7** shows that the large number of parameters in the GrotUNet model is mainly due to the application of the OTblock module, which consists of the Outlooker neighborhood attention layer and the Transformer attention layer. Although the performance of GrotUNet is excellent, it increases the computational complexity and reduces the inference speed, which will be further investigated in the future.

**Table 7:** Ablation study in different configurations of GrotUNet.

Configuration	GRblock	WGRblock	OTblock	R-Skip	Mti-UP	Flops(G)	Parms(M)	FPS	SBD(%)		
									CVPPP	KOMATSUNA	MSU-PID
I	✓	✓	×	✓	✓	21.52	19.14	74.25	86.42	91.54	82.42
II	✓	✓	✓	×	×	44.18	103.87	38.11	87.00	88.6	81.96
III	✓	✓	✓	✓	×	46.13	104.12	42.08	86.62	91.51	81.96
IV	✓	✓	✓	×	✓	46.03	104.21	35.97	85.50	91.21	83.14
V (GrotUNet)	✓	✓	✓	✓	✓	47.99	104.45	36.82	**89.50**	**92.44**	**85.48**

The method in this paper is really experimentally set up for 1×1 convolutional feature aggregation operation in sliding joins. In the experiments, it is found that the performance of segmentation using 1×1 convolution is better than 3×3 convolution, which is beneficial for reducing the number of parameters. In addition, in this paper, the higher-order feature maps are up-sampled by multiscale bilinear interpolation and fused into the shallow decoding sub-networks, and feature aggregation is achieved using the 1×1 convolution operation. Through experiments, it is proved that the multi-scale up-sampling fusion mechanism aggregates the higher-order features with the lower-order features, which effectively improves the quality of the feature maps and preserves more semantic information. Overall, GrotUNet achieves 89.50%, 92.44%, and 85.48% SBD for the evaluation metrics on the CVPPP, KOMATSUNA, and MSU-PID datasets, respectively, which are superior to most of the existing research methods.

## 5 Conclusions

Since plant leaves have overlapping, occluded, and tiny petioles, it is difficult to capture key features using traditional segmentation methods, resulting in inaccurate leaf and petiole detection and poor segmentation performance phenotype. In order to solve the above problems, this paper proposes a novel, end-to-end training leaf segmentation algorithm, GrotUNet. the main contributions of this algorithm are an improved feature extraction encoder, a reconstructed jump connection, and a multiscale upsampling fusion decoder. The encoder consists of three parts: the GRblock, the WGRblock, and the OTblock. The former two utilize the ideas of Resnet and GoogLeNet residual connectivity and parallel branching to fully exploit the semantic features of the image. The latter OTblock, on the other hand, performs one-step mining and encoding of fine-grained image semantic information to obtain finer features. Combining the three effectively extracts the features of local key regions of the instance object. The reconfigured sliding connection module employs a convolutional block at the intermediate node to aggregate semantic information from different scales, which can make the feature representation of each spatial location richer. The decoding backbone network adopts a multi-scale upsampling fusion design to incorporate the outputs of high-level sub-networks into each low-level sub-network, effectively mitigating the loss of semantic information. Experimental evaluations on CVPPP, KOMATSUNA and MSU-PID datasets show that the proposed method GrotUNet outperforms state-of-the-art methods such as UNet, ResUNet, DeepV3+, UNet++, Perspective + UNet. In the future, GrotUNet will be migrated to the fields of crop disease detection and agricultural product quality inspection to further verify its outstanding performance, aiming to provide a strong contribution to the green development of agriculture (Zhenye et al., 2024).

## Data Availability

Publicly available datasets were analyzed in this study. This data can be found here: CVPPP: https://www.plant-phenotyping.org/datasets-home, KOMATSUNA: https://limu.ait.kyushu-u.ac.jp/~agri/komatsuna/, MSU-PID: https://cvlab.cse.msu.edu/category/downloads.html.
